# Climate and Rheumatism
*On Rheumatism, Rheumatic Gout, and Sciatica. By Henry W. Fuller, M.D. Second Edition. London: Churchill. 1856.


**Published:** 1857-07

**Authors:** 


### CLIMATE AND RHEUMATISM.*

Dr. Fuller's very complete and able work on Rheumatism has reached its second edition. Remarking on climate, and its effects on the production of the disease, Dr. Fuller opines that climate *per se* has only a predisposing influence. Looking on the disease as arising from an agent that requires to be eliminated from the body chiefly by the skin, he argues that in summer, when the skin is acting freely, the disorder should be least frequent, and that in damp and foggy seasons it should, for opposite reasons, be most frequent. The facts are so, and the explanation is simple and explicit. The book is an admirable specimen of a standard work, its only defects being its terrific foot-notes, which spoil the reading of the text, and its recipes.

### MISCELLANEOUS WORKS.

A variety of works lie before us, the matter of which has only an indirect bearing on sanitary science. Dr. Dobell has a learned essay on the *Class of Medical Literature most needed in the present day.* It contains some good historical notices. Dr. Webster has an able paper on *Belgian Lunatic Asylums,* which deserves the reading of all who are interested in mental diseases. Dr. Cockle has a good practical essay on the *Second Sound and Murmur of the Heart.* Mr. Acton publishes an essay on the *Reproductive Organs;* the mildest criticism on which is, that other works by the same author may be read with infinitely more profit. Mr. Hare has a book on *Cases of Spinal Deformities,* which is too special to be more than noticed here. Mr. Urling, in a treatise on *Vocal Gymnastics,* recommends imitation of correct sounds as the grand agent in the cure of stammering. Lastly, for time is short, Dr. Fell has brought out, in a *Treatise on Cancer, and its Treatment,* his "original" mode of meeting this fell disease. The spirit of William Shakespeare, without any rapping, left on the reviewer's paper at midnight its criticism on this work:—"Speaks an infinite deal of nothing: his reasons are two grains of wheat hid in two bushels of chaff; you shall seek all day ere you find them; and, when you have them, they are not worth the search." Rather hard on the part of William!

* On Rheumatism, Rheumatic Gout, and Sciatica. By HENRY W. FULLER, M.D. Second Edition. London: Churchill. 1856.

